# Supplementary material for: IdentifiHR predicts homologous recombination deficiency in high-grade serous ovarian carcinoma using gene expression
Source: Commun Med (Lond). 2026 Jan 14;6:119. doi: 10.1038/s43856-026-01387-y (PMC12910048; doi:10.1038/s43856-026-01387-y)
Supplement: Supplementary file 2 — Supplementary Information [file 43856_2026_1387_MOESM2_ESM.pdf]

Supplementary figures and tables

IdentifiHR predicts homologous recombination deficiency in high-grade serous ovarian carcinoma using gene expression.

Ashley L. Weir[1, 2]*, Samuel C. Lee[1, 2], Mengbo Li[1, 2], Ahwan Pandey[3,4], Chin Wee Tan[1, 2, 5], Dale W. Garsed[3,4], Susan J. Ramus[6], Nadia M. Davidson[1, 2]*

1. *The Walter and Eliza Hall Institute, Parkville, VIC 3052, Australia.*
2. *Department of Medical Biology, Faculty of Medicine, Dentistry and Health Sciences, The University of Melbourne, Parkville, VIC 3010, Australia.*
3. *Peter MacCallum Cancer Centre, Melbourne, Victoria 3000, Australia.*
4. *The Sir Peter MacCallum Department of Oncology, The University of Melbourne, Victoria 3010, Australia.*
5. *Frazer Institute, Faculty of Medicine, The University of Queensland, Woolloongabba, Brisbane, QLD 4102, Australia.*
6. *School of Clinical Medicine, UNSW Medicine and Health, University of NSW Sydney, Sydney, NSW 2052, Australia.*

* Co-corresponding authors: davidson.n@wehi.edu.au and weir.a@wehi.edu.au

Supplementary figures

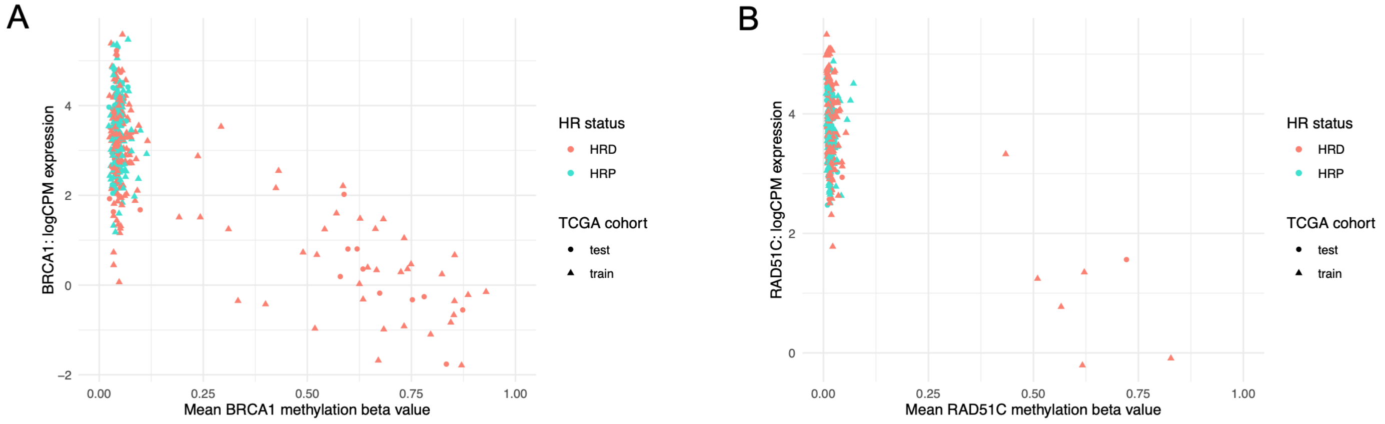

Supplementary figure 1. *The relationship between BRCA1* and *RAD51C promoter methylation and gene expression in the TCGA training and testing cohort (n = 361 HGSCs).* Methylation beta values (A) summarised as the mean beta value for four probes mapping the *BRCA1* promoter (cg04658354, cg10893007, cg19088651, cg19531713) and (B) the exact beta value for a single probe mapping the *RAD51C* promoter (cg14837411), against the matched log_2CPM expression value for each sample of the training (given as triangular points) and testing (given as circular points) cohorts of the TCGA, coloured by HR status.

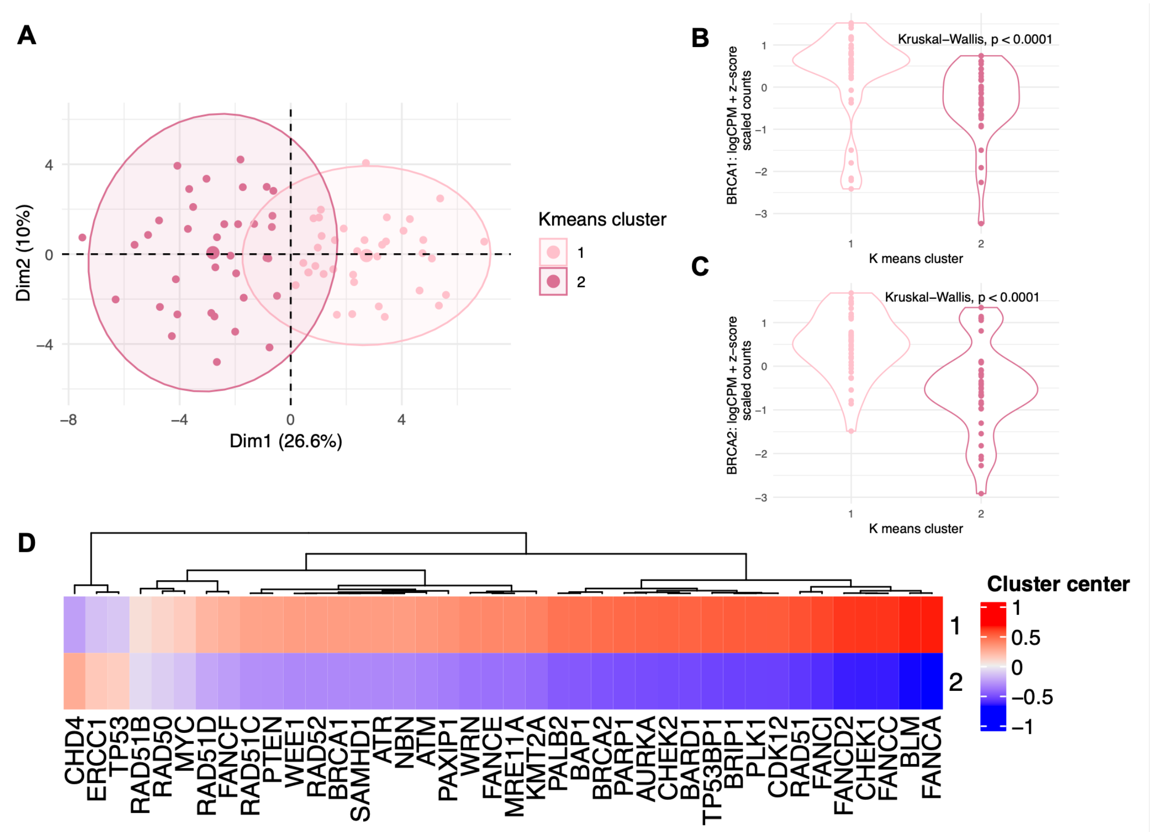

Supplementary figure 2. *K means clustering of the BRCAness gene expression signature in the TCGA testing cohort (n = 73).* (A) Counts of all genes were $\log_2$ CPM transformed, then subset to only genes within the BRCAness signature and z score scaled before being used as input for k means clustering, with 2 centroids. The expression of HR associated genes of the signature, specifically (B) *BRCA1* and (C) *BRCA2* were examined by k means clusters in violin plots, where each point represents a sample, with Kruskal-Wallis testing for expression differences, where significance is defined at $p < 0.05$. (D) The cluster centers of each gene within the signature were also examined to determine the HR status best represented by each cluster.

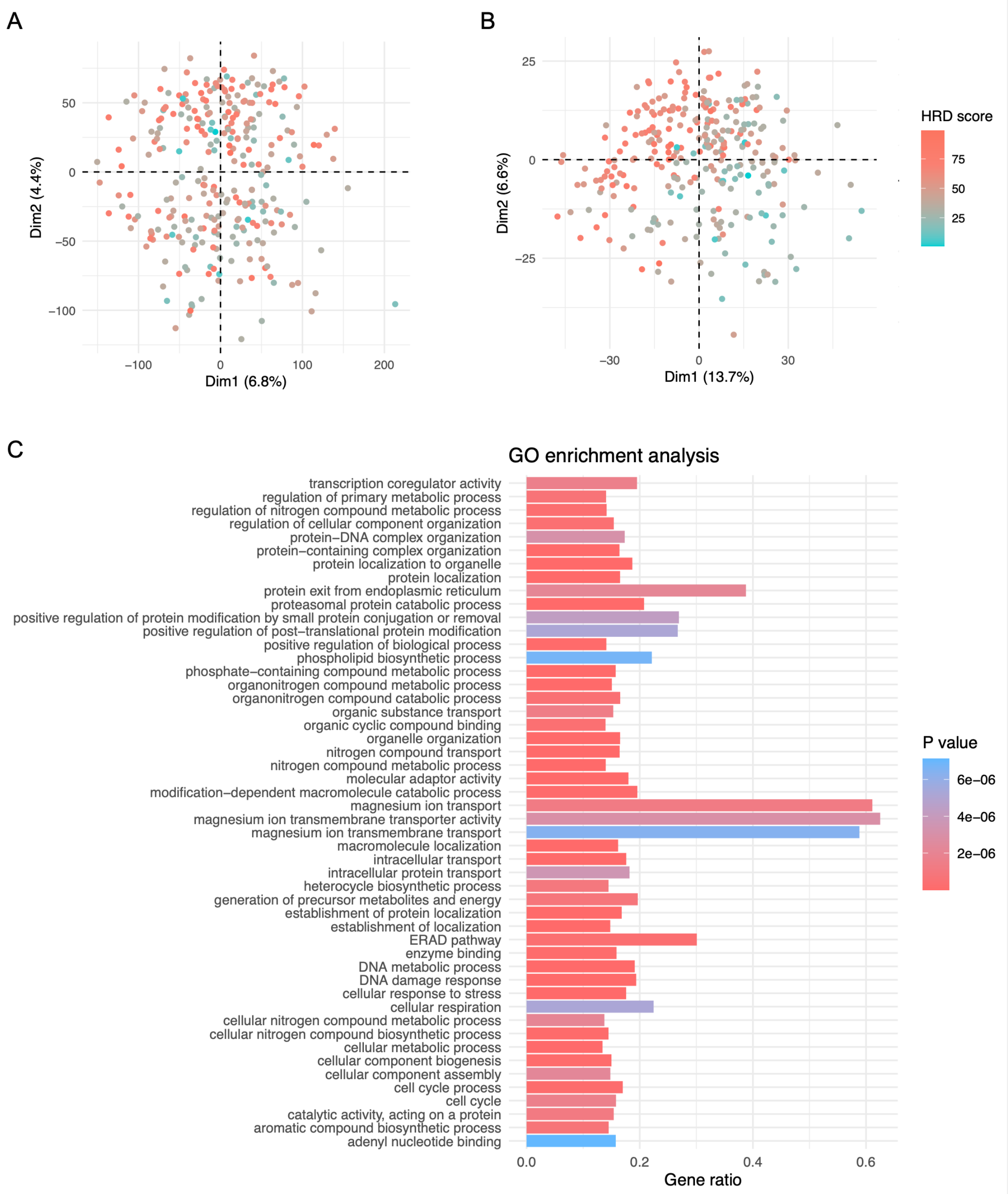

Supplementary figure 3. *Significantly DE genes were used for IdentifiHR feature selection to predict HR status.* PCA plots of the first and second principal components after counts of (A) all genes (n = 52125) and (B) DE genes (n = 2604) were normalised by $\log_2$ CPM and scaled using a z-score. Each point represents a unique sample of the TCGA training cohort (n = 288), coloured by HRD score. (C) The top 50 gene ontologies significantly enriched for differentially expressed genes between HRD and HRP HGSCs. 'Gene ratio' shows the number of DE genes divided by the total number of genes in the ontology set.

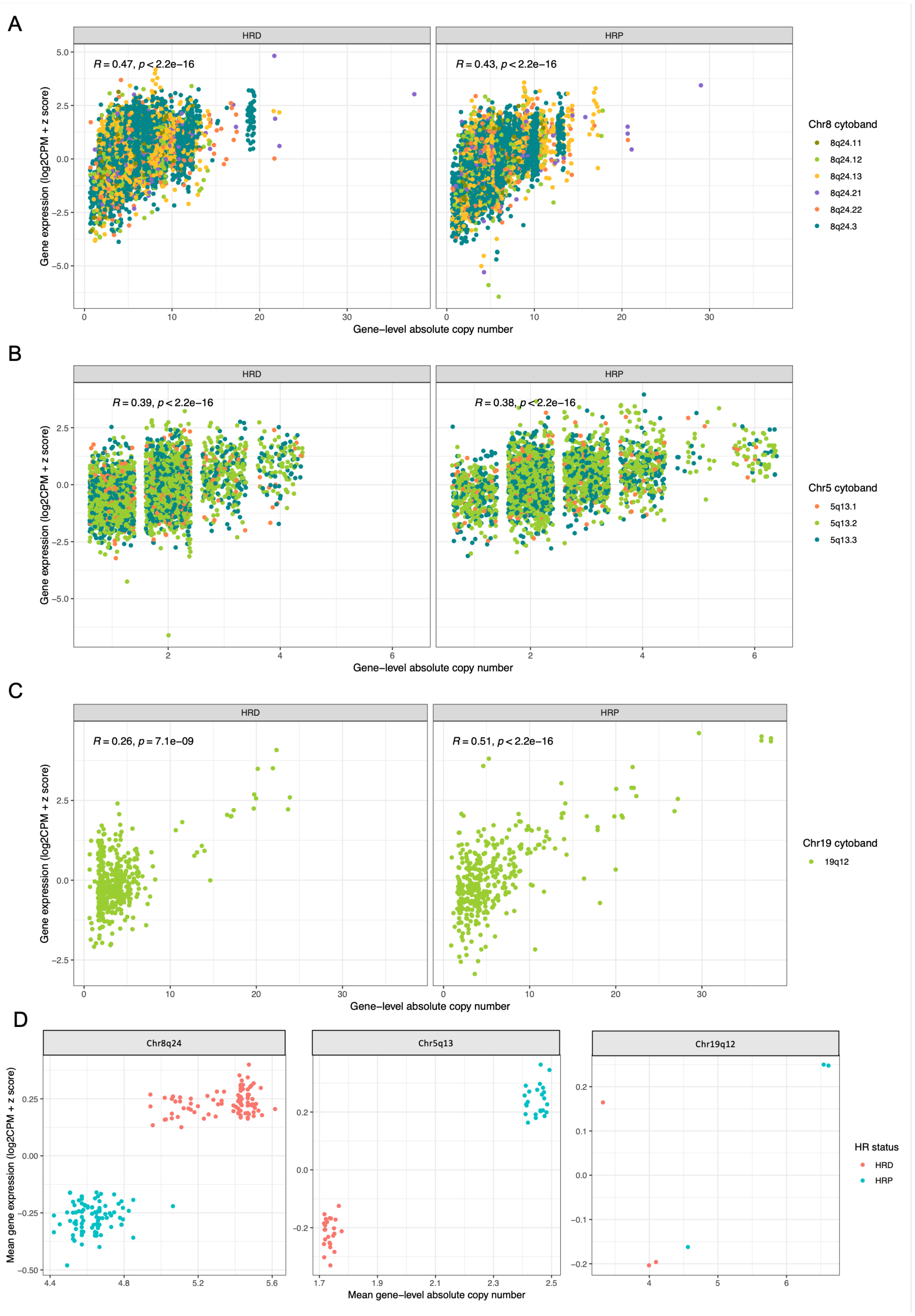

Supplementary figure 4. *The relationship between absolute gene-level copy number and gene expression in specific genomic regions of the TCGA training cohort (n = 288 HGSCs).*

Significantly DE genes in chromosome (A)8q24 (n = 81 genes), (B) 5q13 (n = 24 genes) and (C)19q12 (n = 3), given by log_2CPM transformed and z score scaled expression counts, against the matched absolute copy number for all training cases, by HR status. Each point represents a gene, per HGSC case. Points are coloured by cytoband and the correlation is given by Spearman's R, with the associated p-value. (D) Significantly DE genes as above summarised to the mean expression level, against the matched mean absolute copy number, with points coloured by HR status, across all cases of the TCGA training cohort.

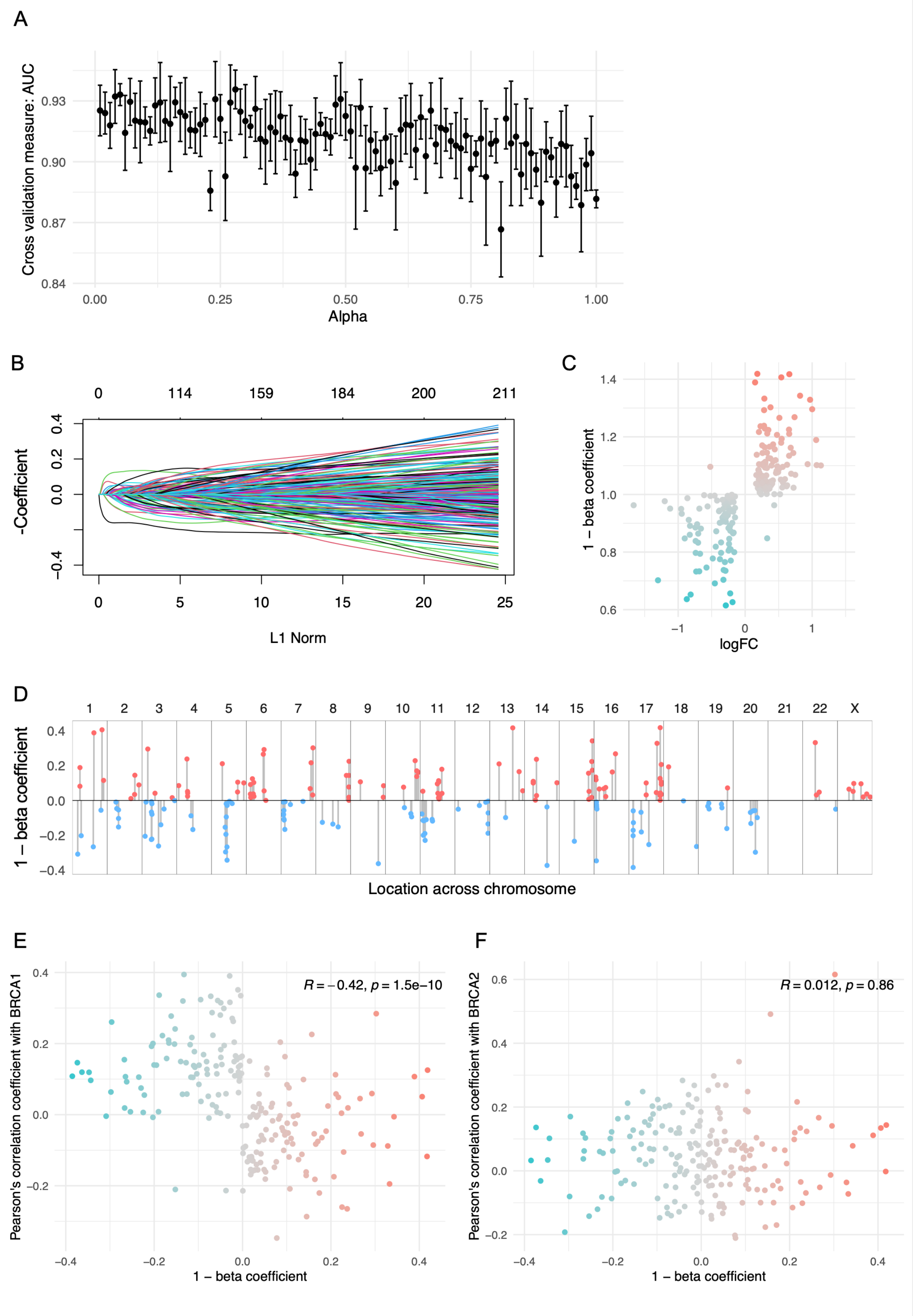

Supplementary figure 5. *209 features were included in the IdentifiHR model, following hyperparameter tuning of alpha and lambda.* (A) Selection of the optimal alpha hyperparameter value, with the associated AUC taken from 5-fold cross validation shown by points, with error bars of 1 standard deviation from each AUC. An alpha of 0.53 was

selected, being the largest alpha value (in the sequence from 0.1 to 1.0, in intervals of 0.1) within 1 standard deviation of the highest AUC for any alpha value. The resulting model required 209 genes as input features; (B) beta coefficients of these features were symmetrically weighted, given by coloured lines, each of which represents a gene in the model. Number of genes at each level of L1 norm given by integers at the top of the plot. (C) Log_2 fold-change (log_2FC) of the 209 weighted genes of IdentifiHR, taken from feature selection DE analysis, against the associated 1- beta coefficient. (D) Chromosomal locations of the 209 model genes given as stemmed points, by the associated 1- beta coefficient that weights the gene in the model. The inverse beta coefficient was visualised for easier comparison with differential expression analysis results. Chromosomes lengths are scaled and given by black borders. The relationship between model weights (1- beta coefficient) for each gene and their respective Pearson's correlation coefficient with (E) *BRCA1* and (F) *BRCA2*, given with the global Pearson's correlation coefficient (R), and associated p-value.

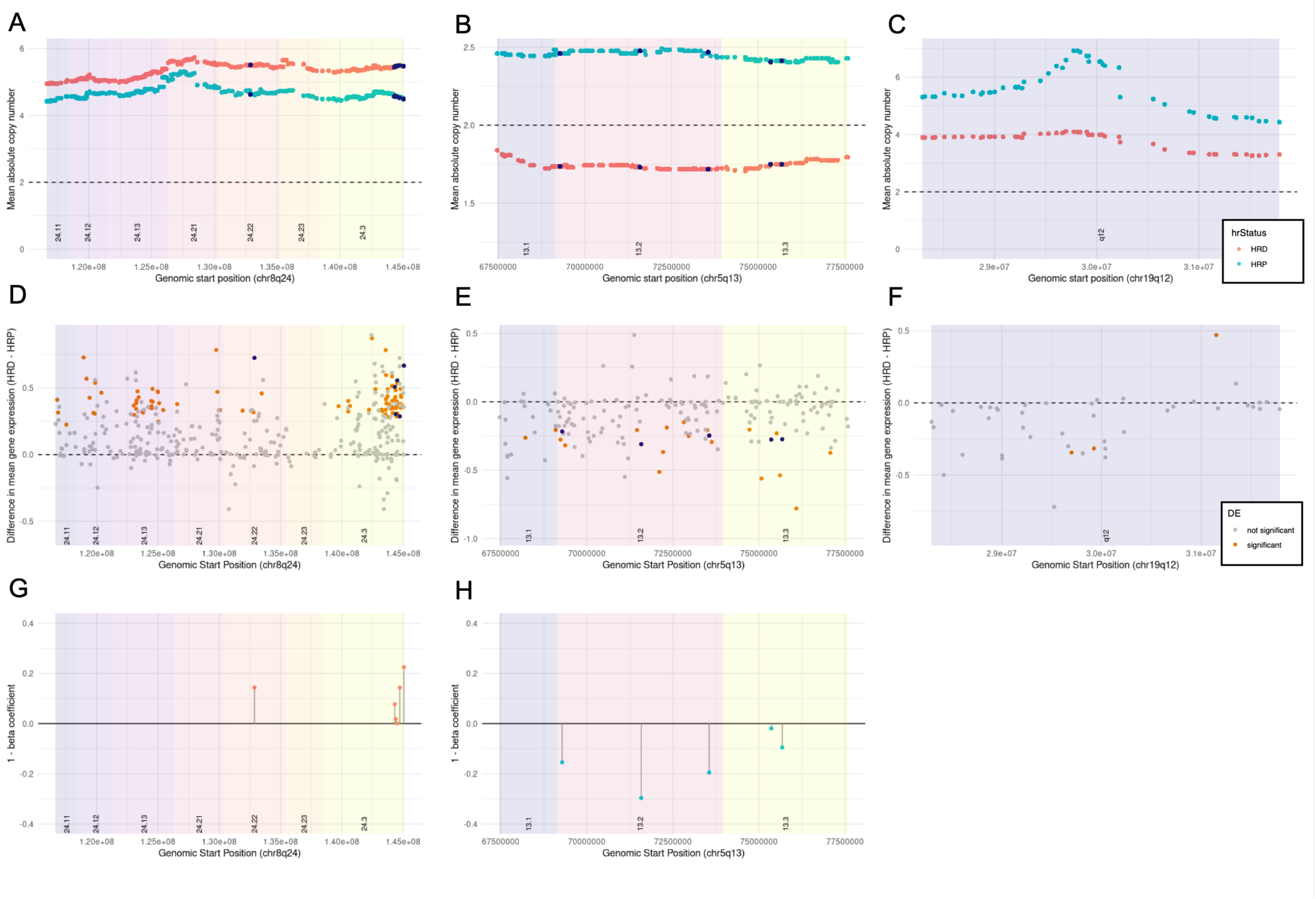

Supplementary figure 6. *The relationship between absolute gene-level copy number, gene expression and IdentifiHR model weights in specific genomic regions of the TCGA training cohort (n = 288 HGSCs).* Genomic regions of interest, include chromosome (A, D, G) 8q24, (B, E, H) 5q13 and (C, F)19q12. Assessed by (A, B, C) the mean absolute gene-level copy number, by HR status, (D, E, F) the difference in mean gene expression (log2CPM) between HRD and HRP cases, coloured by whether differential expression was also significant, and (G, H) IdentifiHR model gene weights (1– beta coefficient), across each cytoband within the region of interest. No genes of 19q12 were weighted in the final model. (A-F) weighted genes of IdentifiHR highlighted navy. Cytobands uniquely coloured within regions and labelled along the x-axis of each plot.

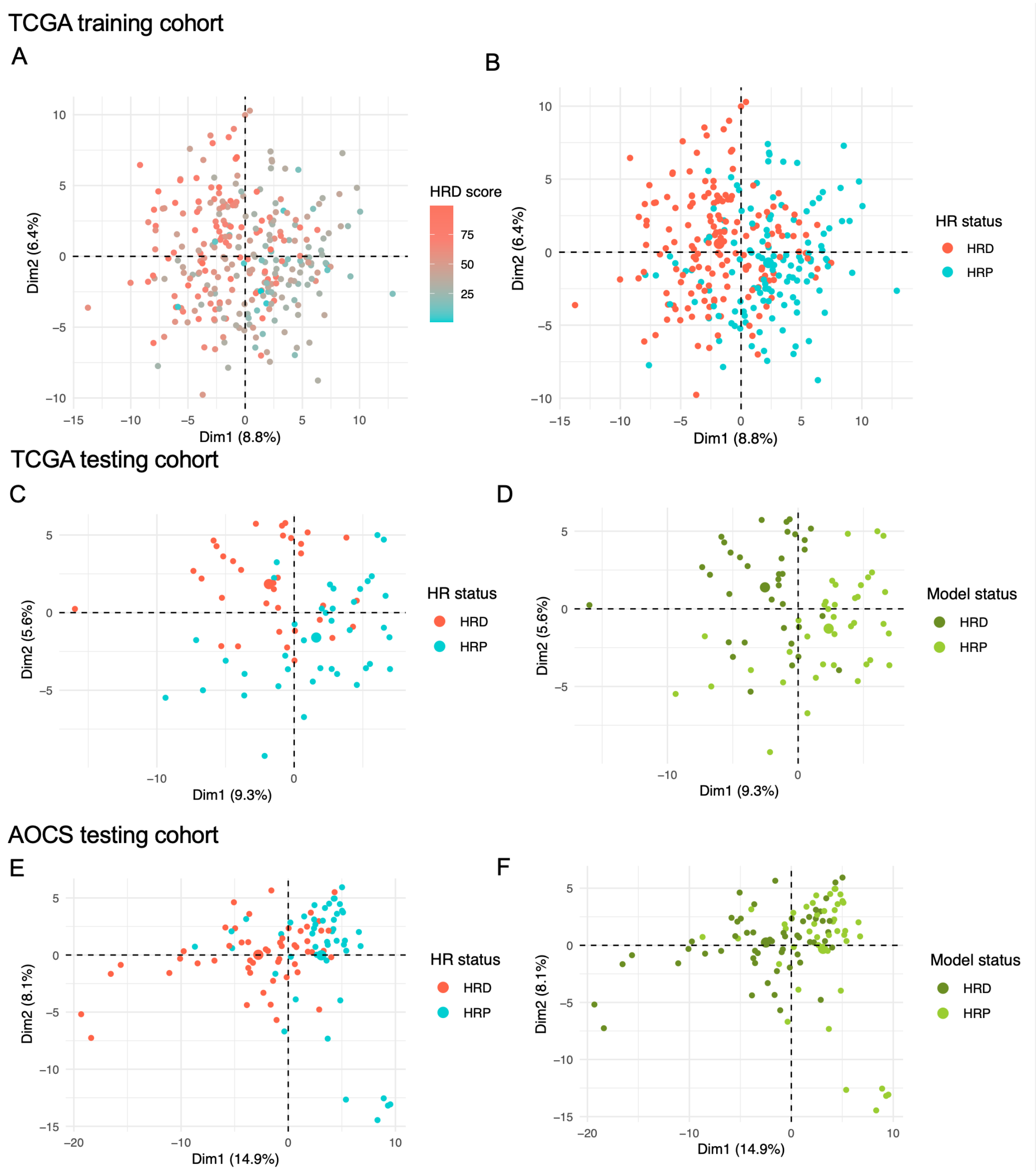

Supplementary figure 7. *HGSCs cluster by the normalised and scaled expression of the 209 genes used by IdentifiHR to predict HR status.* PCA plots of the first and second principal components after counts of IdentifiHR genes (n = 209) were normalised by log2 counts-per-million and scaled using a z-score. Each point represents a unique sample of (A, B) the TCGA training cohort (n = 288), (C, D) the TCGA testing cohort (n = 73) and (E, F) the AOCS testing cohort (n = 99). Points coloured by (A) HRD score and (B, C, E) HR status (HRD defined as having a HRD score of ≥ 42) and (D, F) HR status as predicted by the model.

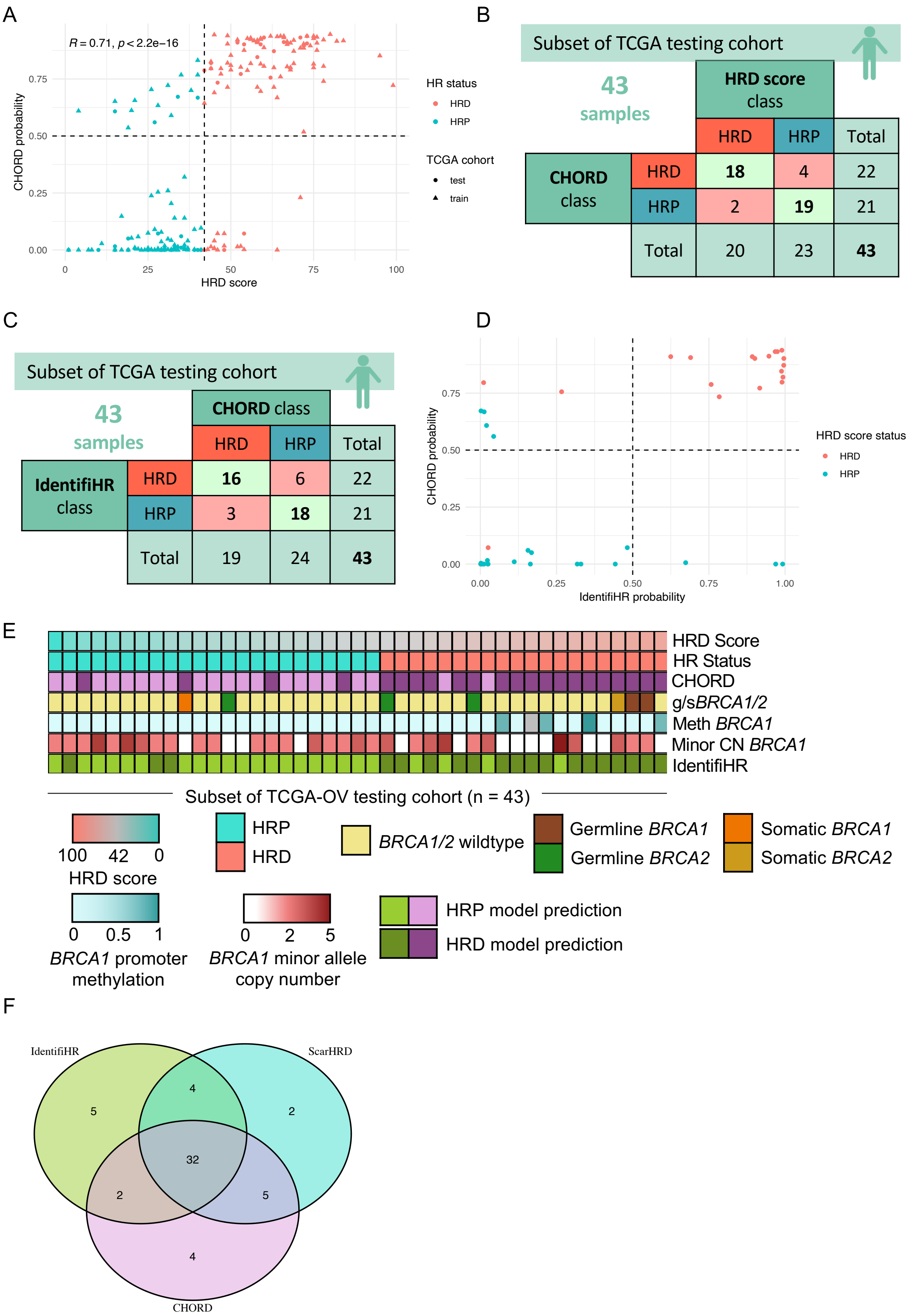

Supplementary figure 8. *Predictions between the HRD score, CHORD and IdentifiHR are concordant in the TCGA HGSC cohort (n = 210 HGSCs).* (A) The relationship between the HRD score, derived from SNP array data, and the probability of a sample being HRD, predicted by CHORD from WGS data, coloured by the HRD score's discrete HR status, in

samples of both the TCGA training and testing cohort, as given by shape. The dashed vertical line at HRD score = 42 indicates the clinical cut-off for HR status classification, and dashed horizontal line indicates the CHORD's predictive cut-off. The Pearson correlation coefficient of a linear fit to the points (R) with the associated p value is also given. Confusion matrix of (B) CHORD's discrete HR status predictions against the genomic HRD score predictions and (C) IdentifiHR predictions against CHORD's discrete HR status predictions in the 43 HGSC cases of the TCGA testing cohort (n = 73) where the required data was present. (D) The relationship between both IdentifiHR's and CHORD's probability predictions for samples being HRD in the subset of the TCGA testing cohort, coloured by the discrete HR status, as defined by HRD score. The dashed lines at model probability = 0.50 indicate the predictive threshold. (E) HRD predictions for each sample (columns) of the subset of the TCGA testing cohort with RNA sequencing, WES and WGS (n = 43). Samples are sorted and coloured by HRD score and HR status (top two tracks) and annotated by CHORD HR status predictions. The presence or absence of germline (g*BRCA1/2*) or somatic (s*BRCA1/2*) mutations in *BRCA1/2* are annotated, with the methylation beta values for the *BRCA1* promoter ("Meth *BRCA1*"), minor allele copy number of *BRCA1* ("Minor CN *BRCA1*") and the HR status as predicted by IdentifiHR are shown. (F)The overlapping concordance in discrete HR status labelling of the three methods.

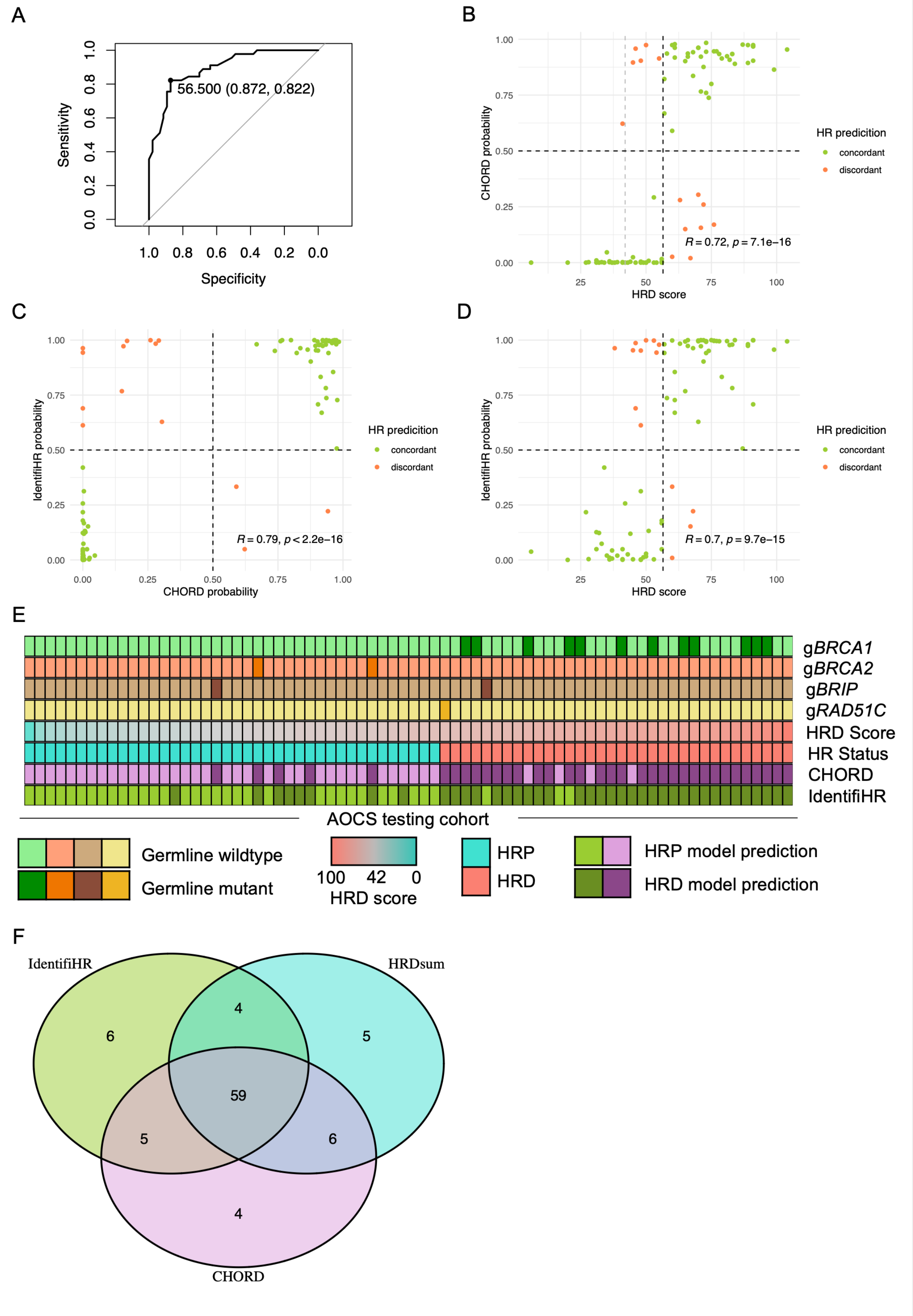

Supplementary figure 9. *IdentifiHR's HR status predictions are concordant with the CHORD prediction and HRDsum score in primary tumours of the AOCS testing cohort (n = 74).* (A) ROC adjustment of the threshold for discrete HR status determination from the HRDsum score, using CHORD HR labels as truth for WGS data of AOCS. Optimal threshold

determined as the value that maximises specificity and sensitivity and printed on curve, with associated specificity and sensitivity. The relationship between (B) HRD score and CHORD probability, (C) CHORD probability and IdentifiHR probability and (D) HRD score and IdentifiHR probability, coloured by the concordance of the two methods if stratified into discrete HR statues. The black dashed lines at HRD score = 56.5 indicates a more appropriate threshold for HR status classification in WGS, as optimised in (A), while the grey dashed line at HRD score = 42 indicates the SNP array threshold for HRD. The dashed lines at CHORD or IdentifiHR probability = 0.50 indicates the model's predictive cut-off. The Pearson correlation coefficient of a linear fit to the points (R) with the associated p value is also given. (E) HR predictions for each primary tumour (columns) of the AOCS testing cohort. Samples are annotated with the presence or absence of germline (g) mutations in *BRCA1/2, BRIP and RAD51C,* sorted and coloured by HRD score and its associated HR status, and detailed with the discrete CHORD and IdentifiHR model predictions of HR status. (F) The overlapping concordance in discrete HR status labelling of the three methods.

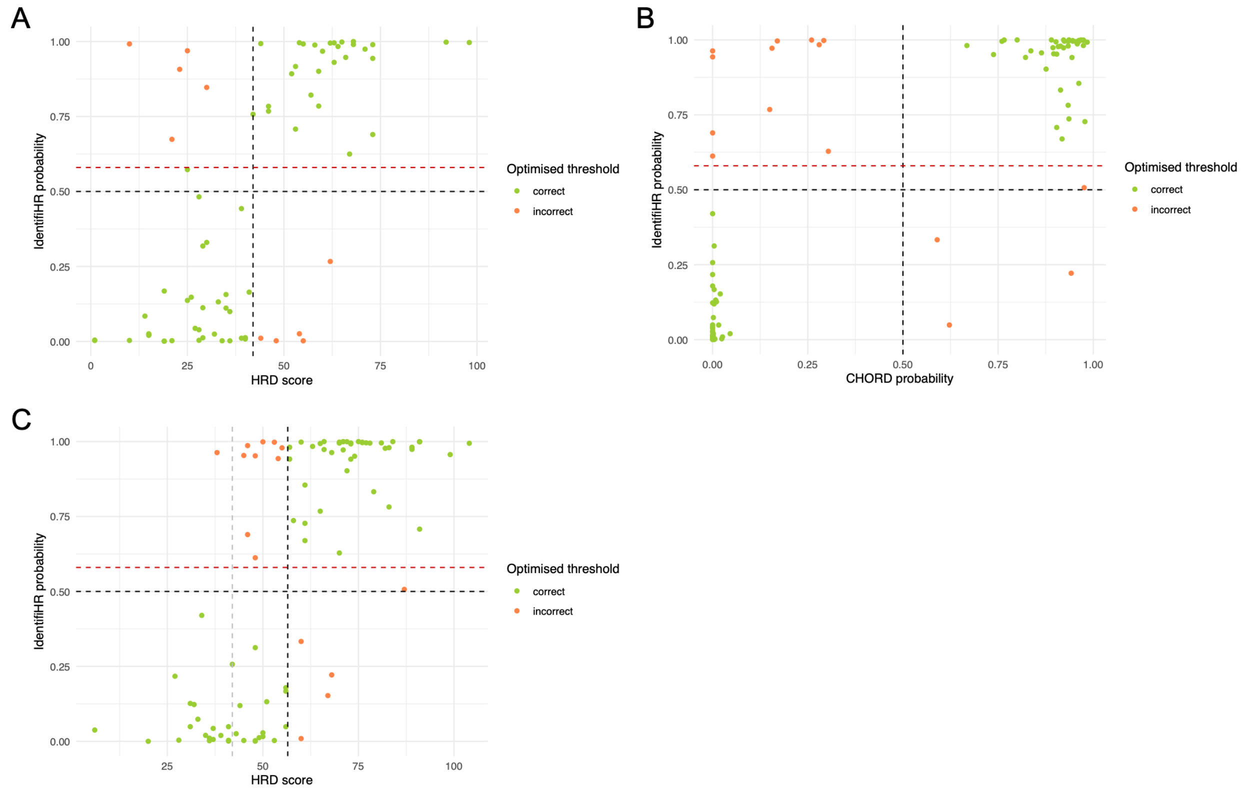

Supplementary figure 10. *Optimising the probability threshold for IdentifiHR in predicting a sample to be HRD, using the TCGA testing cohort.* The relationship between (A) HRD score and IdentifiHR probability in the TCGA testing cohort. The black dashed vertical line at HRD score = 42 indicates the clinical cut-off for HR status classification, using SNP array data. The relationship between (B) CHORD probability and IdentifiHR probability and (C) HRDsum score and IdentifiHR probability in primary tumours of the AOCS testing cohort. The black dashed line at CHORD or IdentifiHR probability = 0.50 indicates the model's predictive cut-off, and at HRD score = 56.5 indicates the ROC-adjusted threshold for HR status classification, while the grey dashed line at HRD score = 42 represented the threshold for SNP array data. Points coloured by the concordance of the two methods after applying the optimised probability threshold of 0.58, as opposed to 0.50, to stratify cases into discrete HR statues. The red dashed horizontal line at IdentifiHR probability = 0.58 indicates the optimised threshold from the TCGA testing cohort for a sample being HRD.

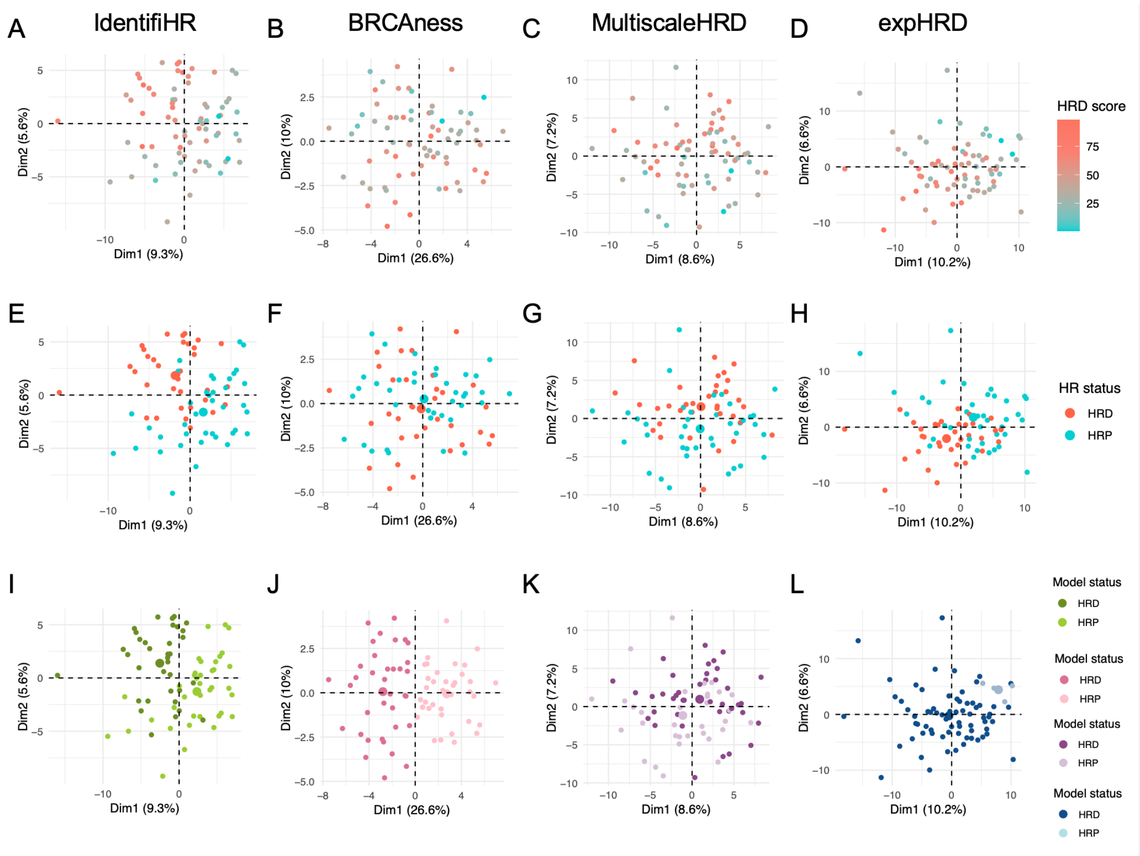

Supplementary figure 11. *Qualitatively comparing gene-expression based tools to predict HR status using PCA.* PCA using the normalised and scaled input genes for (A, E, I) IdentifiHR (n = 209 genes), (B, F, J) BRCAness (n = 40 genes), (C, G, K) MultiscaleHRD (n = 228 genes) and (D, H, L) expHRD (n = 356 genes) in the TCGA HGSC testing cohort (n = 73 samples). Samples are clustered by (A, B, C, D) HR status, (E, F, G, H) HRD score and (I, J, K, L) the predicted HR status given by each model.

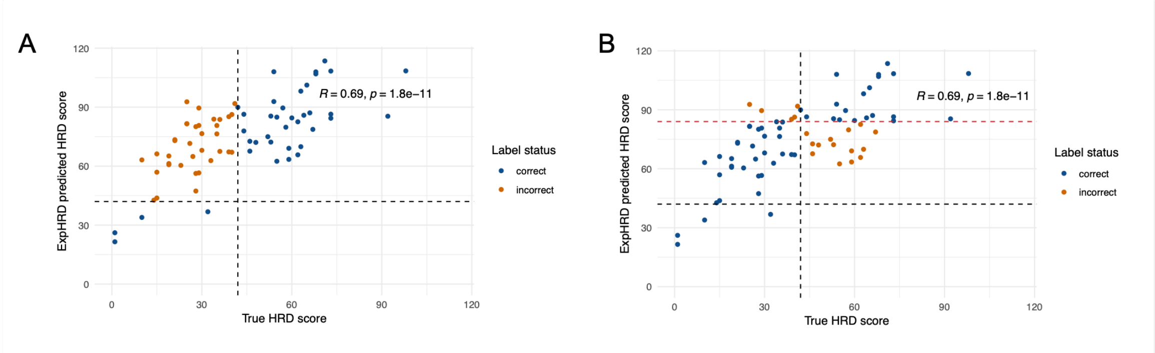

Supplementary figure 12. *The relationship between the true HRD score and the HRD score predicted by the expHRD model in the TCGA testing cohort (n = 73 samples).* The black horizontal and vertical dashed lines at (A) 42, show the clinical HRD score cut-off that separates HRP and HRD and (B) the red horizontal dashed line at 84, to demonstrate the optimal cut-off to maximise model accuracy. Points coloured by whether expHRD predicted the sample's HR status correctly or incorrectly, at each cut-off.

Supplementary tables

Supplementary table 1. Evaluating the proportion of each HGSC FIGO stage across HRD and HRP cases of the training and testing cohort from the TCGA respectively.

	Training		Testing	
**FIGO stage**	**HRP**	**HRD**	**HRP**	**HRD**
Stage IC	0	1	0	0
Stage IIA	3	0	0	0
Stage IIB	1	2	0	0
Stage IIC	3	9	1	1
Stage IIIA	2	3	0	1
Stage IIIB	5	7	1	1
Stage IIIC	92	110	32	28
Stage IV	20	27	5	3
Stage unknown *	2	1	0	0
p value[a]	0.50		0.84	

* Cases with unknown stage were not included in statistical testing

[a] Chi-squared goodness-of-fit test performed on training and testing cases separately.

Supplementary table 2. Evaluating the proportion of each race across HRD and HRP cases of the training and testing cohort from the TCGA respectively.

	**Training**		**Testing**	
**Race**	**HRP**	**HRD**	**HRP**	**HRD**
American Indian or Alaska native	0	1	0	1
Asian	4	4	0	2
Black or African American	7	12	4	2
Native Hawaiian or other pacific islander	0	1	0	0
White	115	137	33	27
Race unknown *	2	5	2	2
p value[a]	0.70		0.41	

* Cases with unknown stage were not included in statistical testing

[a] Chi-squared goodness-of-fit test performed on training and testing cases separately.
