# Supplementary material for: IdentifiHR predicts homologous recombination deficiency in high-grade serous ovarian carcinoma using gene expression
Source: Commun Med (Lond). 2026 Jan 14;6:119. doi: 10.1038/s43856-026-01387-y (PMC12910048; doi:10.1038/s43856-026-01387-y)
Supplement: Supplementary file 3 — Description of Additional Supplementary Files [file 43856_2026_1387_MOESM3_ESM.pdf]

Description of Additional Supplementary Files

Supplementary Data 1- Training and testing cohort split in TCGA. Sample identifiers and their assignment to either the training or testing cohort of this manuscript

Supplementary Data 2- DE genes when contrasting HRD - HRP. Top table of differentially expressed genes, contrasting HRD - HRP in the TCGA HGSC training cohort. Differential expression of genes following linear modelling fitting and t-statistic calculation by robust empirical Bayes moderation of standard errors towards a common value. Estimated log2 fold-change (logFC) in gene expression corresponding to the contract HR deficient – HR proficient. Benjamini-Hochberg adjusted p value.

Supplementary Data 3- Gene ontology results. Gene ontology enrichment analysis by goana, on the top table of differential gene expression, contrasting HRD - HRP HGSCs

Supplementary Data 4- The 209 genes of identifiHR. Genes included as input for IdentifiHR's prediction of HR status in HGSC, associated beta coefficients, and their correlation with BRCA1/2.

Supplementary Data 5- HR status predicitions of TCGA testing cohort using IdentifiHR. TCGA testing cohort predictions of HR status using IdentifiHR, given as discrete classes and probabilities.

Supplementary Data 6- HR status predicitions of AOCS testing cohort using IdentifiHR. AOCS testing cohort predictions of HR status using IdentifiHR, given as discrete classes and probabilities.

Supplementary Data 7- HR status predicitions of pseudobulked HGSC cells of the MSKCC - Pt testing cohort using IdentifiHR. MSKCC - Pt testing cohort, whereby HGSC have been pseudobulked at the patient level, to give predictions of HR status using IdentifiHR, given as discrete classes and probabilities.

Supplementary Data 8- HR status prediciton accuracy by sample site in MSKCC - Ss testing cohort using IdentifiHR. Confusion matrix of HR status predicitions in HGSC cells pseudobulked at the site and patient level of the MSKCC testing cohort using IdentifiHR

Supplementary Data 9- HR status predicitions of pseudobulked HGSC cells of the MSKCC - Ss testing cohort using IdentifiHR. MSKCC - Ss testing cohort, whereby HGSC have been pseudobulked at the patient and sample site level, to give predictions of HR status using IdentifiHR, given as discrete classes and probabilities.
